# Does Last Year’s Cost Predict the Present Cost? An Application of Machine Leaning for the Japanese Area-Basis Public Health Insurance Database

**DOI:** 10.3390/ijerph18020565

**Published:** 2021-01-12

**Authors:** Yoshiaki Nomura, Yoshimasa Ishii, Yota Chiba, Shunsuke Suzuki, Akira Suzuki, Senichi Suzuki, Kenji Morita, Joji Tanabe, Koji Yamakawa, Yasuo Ishiwata, Meu Ishikawa, Kaoru Sogabe, Erika Kakuta, Ayako Okada, Ryoko Otsuka, Nobuhiro Hanada

**Affiliations:** 1 Department of Translational Research, Tsurumi University School of Dental Medicine, Yokohama 230-8501, Japan; ishikawa-me@tsurumi-u.ac.jp (M.I.); sogabe-k@tsurumi-u.ac.jp (K.S.); otsuka-ryoko@tsurumi-u.ac.jp (R.O.); hanada-n@tsurumi-u.ac.jp (N.H.); 2 Ebina Dental Association, Kanagawa 243-0421, Japan; ishiiryo141@gmail.com (Y.I.); yota@db3.so-net.ne.jp (Y.C.); shun-s@wg8.so-net.ne.jp (S.S.); suzuki@bell-dental.com (A.S.); lion@kd5.so-net.ne.jp (S.S.); morita-d-c-2@t06.itscom.net (K.M.); tanabedental5@me.com (J.T.); cherry@cherry-dental.com (K.Y.); yasuo-i@rb3.so-net.ne.jp (Y.I.); 3 Department of Oral Microbiology, Tsurumi University School of Dental Medicine, Yokohama 230-8501, Japan; kakuta-erika@tsurumi-u.ac.jp; 4 Department of Operative Dentistry, Tsurumi University School of Dental Medicine, Yokohama 230-8501, Japan; okada-a@tsurumi-u.ac.jp

**Keywords:** healthcare cost, medical healthcare cost, dental healthcare cost, zero-inflated model, neural network

## Abstract

The increasing healthcare cost imposes a large economic burden for the Japanese government. Predicting the healthcare cost may be a useful tool for policy making. A database of the area-basis public health insurance of one city was analyzed to predict the medical healthcare cost by the dental healthcare cost with a machine learning strategy. The 30,340 subjects who had continued registration of the area-basis public health insurance of Ebina city during April 2017 to September 2018 were analyzed. The sum of the healthcare cost was JPY 13,548,831,930. The per capita healthcare cost was JPY 446,567. The proportion of medical healthcare cost, medication cost, and dental healthcare cost was 78%, 15%, and 7%, respectively. By the results of the neural network model, the medical healthcare cost proportionally depended on the medical healthcare cost of the previous year. The dental healthcare cost of the previous year had a reducing effect on the medical healthcare cost. However, the effect was very small. Oral health may be a risk for chronic diseases. However, when evaluated by the healthcare cost, its effect was very small during the observation period.

## 1. Introduction

The increasing healthcare cost imposes a large economic burden for the Japanese government. In Japan, the national insurance system covers a wide range of treatment of diseases and injuries including dental treatment and medication. By the annual report of “Estimates of National Medical Care Expenditure, FY 2017”, which summarizes the expense of the national health insurance, the total healthcare cost was JPY 43.0710 trillion with a 2.2% increase on the previous fiscal year. It occupied 7.78% of the GDP and 10.66% of the national income. The per capita cost was JPY 339,900. Twenty-five percent of the source funding was from the national treasury and 13% from local governments [1].

Oral diseases are the most prevalent diseases globally and have serious health and economic burdens. The most frequent disease leading to death worldwide is a non-communicable disease. Oral health, especially the periodontal condition has been suggested to be affected by noncommunicable diseases [2,3,4,5,6,7,8,9,10,11]. An imbalance towards a periodontal immune response is underlined for other chronic diseases [11]. Epidemiological studies had shown that periodontitis was associated with the metabolic syndrome [3] and cardiovascular disease [4,7]. The suboptimal oral function was a potential risk of mortality [12,13]. Periodontitis was associated with an increased risk of all-cause mortality, mortality due to cardiovascular diseases, cancer, coronary heart disease, and cerebrovascular diseases [2]. The prevention and intervention of oral disease may lead to improving the health status and is finally expected to lead to reducing the medical healthcare cost.

In the previous report, statistical models were constructed to predict the healthcare cost by the periodontal status and dental healthcare cost [14,15]. The limitation of these studies were a small sample size and the subjects analyzed in these studies were adults who engaged in a specific occupation: High school teachers [14] and the clerk of the insurance company [15].

In contrast to previous studies, the area-basis public health insurance database contains a large sample size and subjects and their families engaged in a variety of occupations. By analyzing the area-basis public health insurance database, a more validated and general statistical model to evaluate the effect of oral health on the healthcare cost can be constructed.

In this study, a database of the area-basis public health insurance of one city was analyzed. The aim of this study was to present the descriptive statistics of the healthcare cost and to predict the medical healthcare cost by the dental healthcare cost.

## 2. Materials and Methods

### 2.1. Setting

Ebina city is located in Kanagawa Prefecture next to Tokyo, Capital of Japan. The population of Ebina city was 135,619 at 1 October 2020. Ninety-one subjects were born and 84 died during October 2020. Four hundred and seventy-two subjects were moved in and 440 subjects were moved out during October 2019. A total of 36,856 subjects were subscribed to the area-basis public health insurance of Ebina city at 1 April 2017 [16].

### 2.2. Database

The Japan Federation of National Health Insurance Organization constructed and provided the database software for all the cities or villages that managed the area-basis public health insurance. This database is called the Kokuho database. The abbreviation of “Koku” means nation and “ho” means insurance. This database is conventionally known as KDB. KDB summarizes the monthly healthcare cost per capita. Healthcare costs are summarized by the medical healthcare cost, medication cost, and dental healthcare cost. The medical healthcare cost was summarized separately by the hospitalized patients care cost and outpatients care cost. The data from April 2017 to September 2018 were completed in October 2020. In this study, the data during this duration were analyzed by the exported CSV file.

### 2.3. Statistical Modeling

#### 2.3.1. Generalized Linear Model and Zero-Inflated Model

Before the application of the zero-inflated model, the medical and dental costs were categorized. Optimal categorizations were performed by the SPSS Statistics version 24.0 (IBM, Tokyo, Japan).

For the prediction of the medical healthcare cost, the generalized linear model and zero-inflated models were constructed [17]. The categorized medical healthcare cost was used as a dependent variable. The age, sex, categorized medical healthcare cost of the previous year, and the categorized dental healthcare cost of the previous year were used as an independent variable. The model fit was evaluated by Akaike’s information criteria (AIC). To improve the model fit, link functions and distributions were changed. The model that had the least AIC was selected. The R software version 3.50 with AER, glm2, pscl, and MASS package was used.

#### 2.3.2. Neural Network Model

The multilayer perceptron was applied to predict the medical healthcare cost [14,15,18]. The age, sex, medical healthcare cost of the previous year, and dental healthcare cost of the previous year were used as predictor values. The data were randomly divided into 15 groups. One group was used for the construction of the model and the model was trained by the other data of the 14 groups. The model construction and prediction were performed by the SPSS Modeler version 18.22 (IBM, Tokyo, Japan).

#### 2.3.3. Support Vector Machine Regression

Support vector machine regression was performed by the R software with e1071 and the kernlab package. The model was constructed by the spline kernel as a kernel function. A four times-fold cross validation on the training data were used for training the model.

#### 2.3.4. Generalized Boosted Regression Models

Generalized boosted regression models were constructed by the R software with the gbm package and the following parameter settings: Distribution = “gaussian”, n.trees = 100, shrinkage = 0.1, interaction.depth = 3, bag.fraction = 0.5, train.fraction = 0.5, n.minobsinnode = 10, cv.folds = 5.

### 2.4. Ethics

The study protocol was approved by the Ethical Committee of Tsurumi University School of Dental Medicine (approval number: 1747).

## 3. Results

### 3.1. Descriptive Statistics of the Healthcare Cost

**Figure 1:**
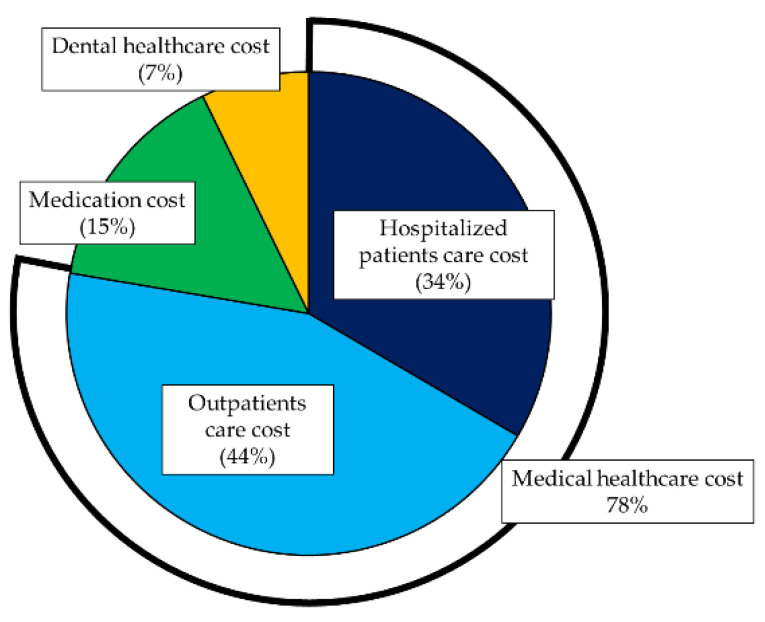
Total healthcare cost during 2017 to the first half of 2018.

From April 2017 to September 2018, 6526 subjects were resigned from the area-basis national health insurance of Ebina city and 2901 subjects were newly affiliated. A total of JPY 14,899,646,550 were used as the healthcare cost during this period. The 30,340 subjects who had continued registration of the area-basis public health insurance of Ebina city were analyzed. The study population consisted of 15,787 men and 14,553 women, and their age was 53.23 +/− 19.60 for the mean and standard deviation, and 62 (41–69) for the median and 25th to 75th percentile. The healthcare cost of these subjects were JPY 13,548,831,930. The per capita healthcare cost was JPY 446,567. The itemized healthcare cost is shown in Figure 1. The medical healthcare cost, which is the sum of hospitalized patients care cost and outpatients care cost, was 78% of the total cost and the dental healthcare cost was 7%. The itemized healthcare cost by the age groups was shown in Table S1. The distribution of the healthcare cost was skewed. During this one year and half period, 5707 (18.8%) subjects did not use a medical service, and 10,336 (34.1%) did not use a medication service, and 14,717 (48.5%) did not used a dental service.

The numbers indicate the sum of each cost during 2017 to the first half of 2018 (Japanese yen). The medical healthcare cost is the sum of hospitalized patients care cost and outpatient care cost. The itemized national healthcare cost was JPY 4,531,692,560 (37.6%) for the hospitalized patients care cost, JPY 5,992,460,680 (33.9%) for the outpatients’ care cost, JPY 2,047,271,910 (18.1%) for the medication cost, and JPY 977,406,780 (6.7%) for the dental healthcare cost. The proportion of the itemized healthcare cost was not statistically significant when compared with the proportion of the national healthcare cost by the chi-square test (*p* = 0.630).

### 3.2. Healthcare Cost by Specific Diseases

**Figure 2:**
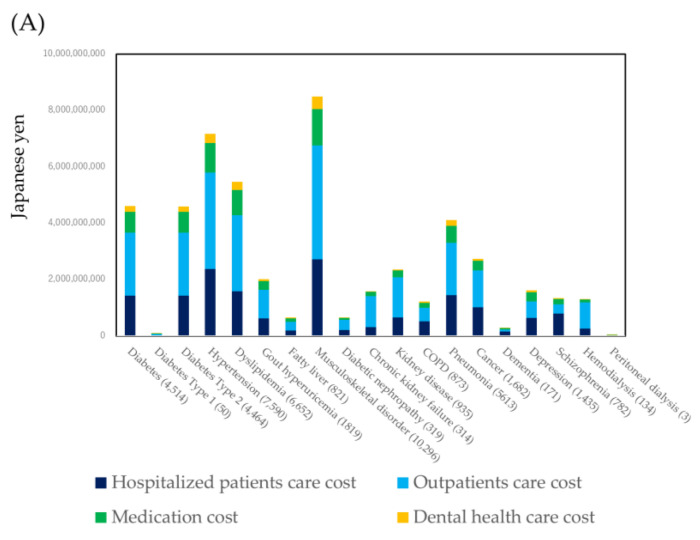

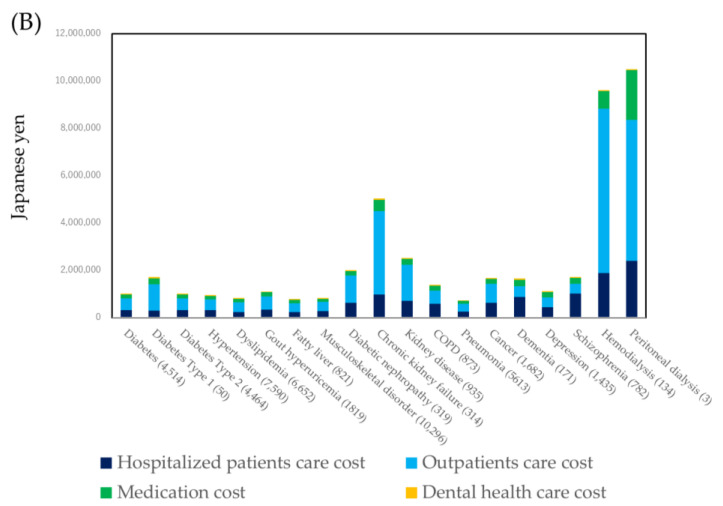
Healthcare cost of specific diseases. (**A**) Total healthcare cost, (**B**) healthcare cost per subjects.

The KDB database contained a diagnosis of major diseases. For these diseases, the total healthcare cost during one year and half and the healthcare cost per capita were illustrated by a bar chart (Figure 2). For the total healthcare cost, musculoskeletal disease was highest followed by hypertension. For the healthcare cost per capita, peritoneal dialysis was highest followed by hemodialysis. However, the number of patients with peritoneal dialysis and hemodialysis were a very small fraction.

The KDB database contained a diagnosis of major diseases and injuries. The healthcare cost by a specific disease was summarized by the sum of the healthcare cost during a one and half year period (A) and per capita during a one and a half year period (B).

### 3.3. Prediction of Medical Healthcare Cost

#### 3.3.1. Descriptive Analysis

**Figure 3:**
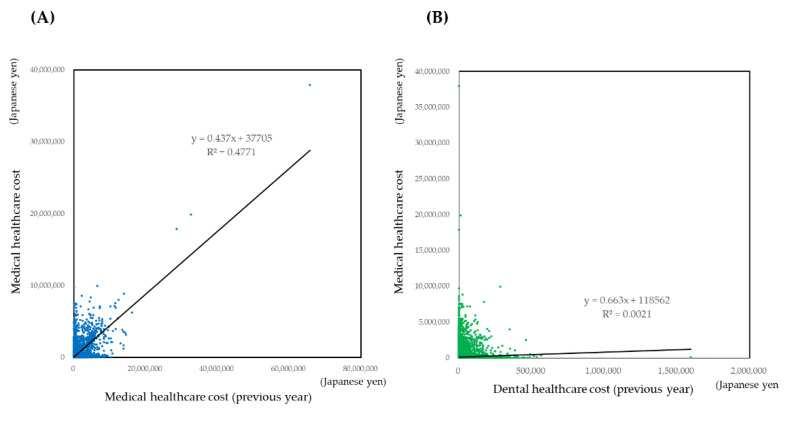

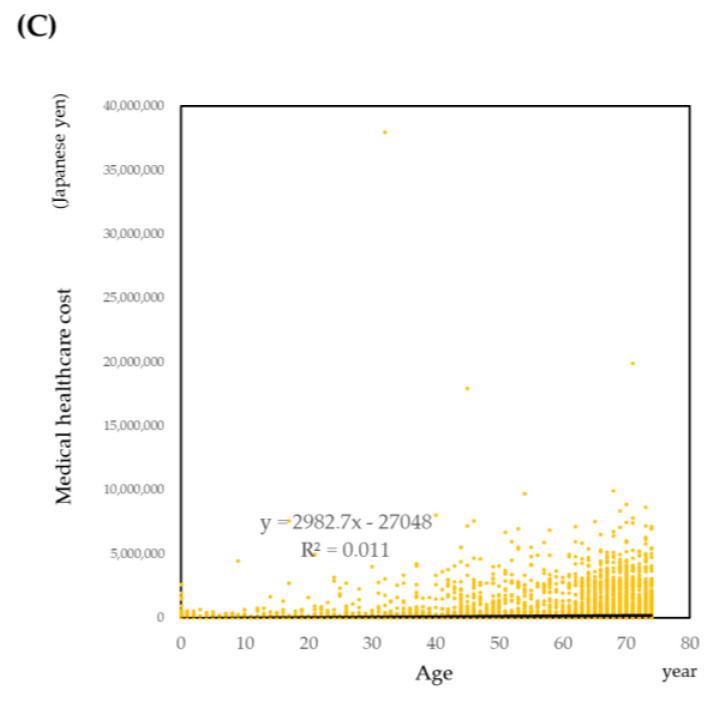
Scatter plot of medical healthcare cost against the medical healthcare cost of the previous year (**A**), dental healthcare cost of the previous year (**B**), and age (**C**). Plots were aggregated on the *x* or *y* axis.

The scatter plot of medical cost against the medical cost of the previous year and dental cost of the previous year were shown in Figure 3. Plots were aggregated on the *x* and *y* axis. The regression lines by simple regression were not reliable. Therefore, to estimate the medical cost by the medical cost or dental cost of the previous year, statistical modeling is indispensable.

#### 3.3.2. Prediction of Medical Healthcare Cost by Regression Models

The medical healthcare cost during the half year was estimated by the regression model. The age, sex, medical healthcare cost of the previous year, and dental healthcare cost of the previous year were used as an independent variable. The conventional generalized linear model and zero-inflated model were applied (Table 1). When compared by Akaike’s information criteria, the fittest model was the zero-inflated model. The zero-inflated model consisted of two components: Zero-inflation model and count model. For the zero-inflation model, the coefficient of the medical healthcare cost and dental healthcare cost of the previous year were negative and statistically significant. It indicated that subjects who used the medical or dental healthcare cost of the previous year tended not to use the medical healthcare cost the next year. By the count model, the coefficient of medical healthcare cost was positive and statistically significant. It indicated that the amount of the medical healthcare cost positively depended on the amount of the medical healthcare cost of the previous year. The coefficient of the dental healthcare cost was not statistically significant. It indicated that the amount of the medical healthcare cost was not dependent on the amount of dental healthcare cost of the previous year. For the prediction of the medical healthcare cost, the zero-inflated model gave us significant factors. However, there is a limitation for the zero-inflated model. Variables need to be treated as discrete variables.

#### 3.3.3. Prediction of Medical Healthcare Cost by the Neural Network Model, Support Vector Machine Regression, and Generalized Boosted Regression Modeling (GBM)

**Figure 4:**
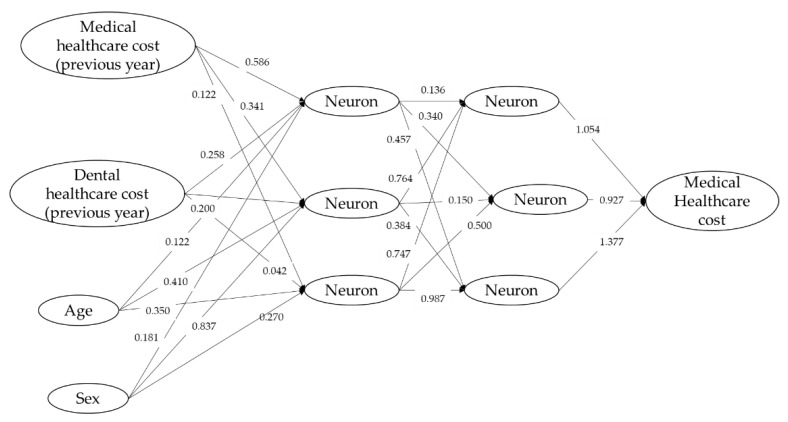
Neural network model for the prediction of medical healthcare cost.

**Figure 5:**
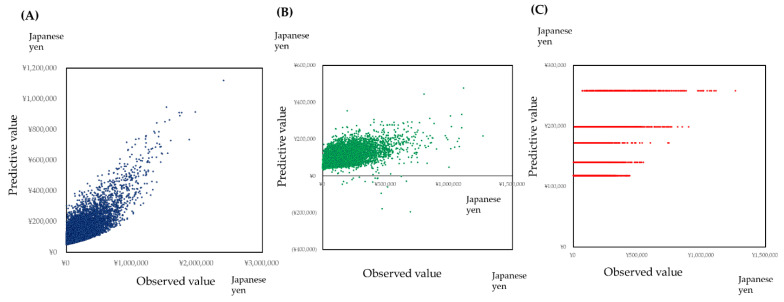
Predictive performance of the neural network model (**A**), support vector machine regression (**B**), and generalized boosted regression modeling (**C**). Predictive performance was shown by the scatter plot of the predictive value against the observed value.

**Figure 6:**
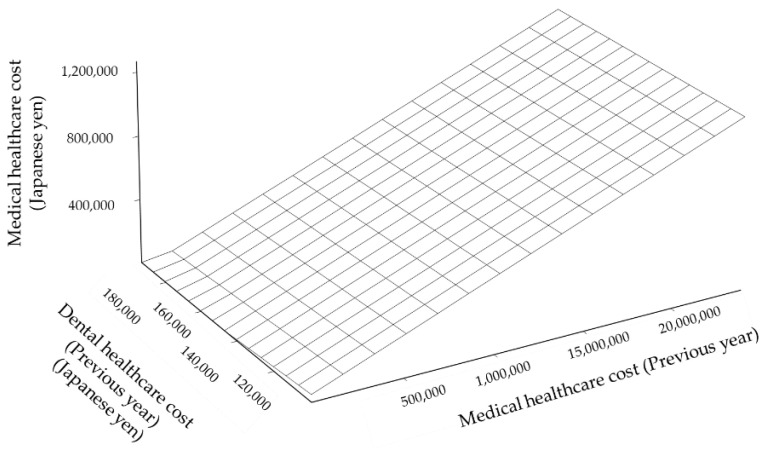
Response surface of the prediction of the medical healthcare cost by the medical healthcare cost of the previous year and dental healthcare cost of the previous year.

Since the zero-inflated model has limitations for high values, the neural network model, support vector machine regression, and generalized boosted regression modeling (GBM) were applied to predict the medical healthcare cost. The age, sex, medical healthcare cost, and dental healthcare cost of the previous year were used as independent variables. The constructed neural network model was shown in Figure 4. The errors of square sum were 6687 for the learning step and 5626 for the test step. The predictive performances of these models are shown by the scatter plot of predictive values against the observed value: Neural network model (Figure 5A), Support Vector Machine Regression Figure 5B), and GBM (Figure 5C). The response surface of the prediction of the medical healthcare cost is shown in Figure 6. The medical healthcare cost proportionally depended on the medical healthcare cost of the previous year. The dental healthcare cost of the previous year had a reducing effect on the medical healthcare cost. However, the effect was very small. The information of the support vector machine regression and generalized boosted modeling were shown in Table S2 and Figure S1. 

## 4. Discussion

The prediction of the healthcare cost is important for health policy making to decide the priority for the prevention of the disease. In this study, the medical healthcare cost was predicted by the medical healthcare cost of the previous year and the dental healthcare cost of the previous year. Our previous report had shown that conventional regression models were not applicable for the prediction of the medical cost [14,15].

The proportion of the itemized healthcare cost was not statistically significant when compared with the national healthcare cost. It indicated that the data analyzed in this study represented the national healthcare cost of Japan (Figure 1).

Some studies tried to predict the healthcare cost by the statistical modeling [19,20,21,22,23,24]. The age, gender, and a previous year’s healthcare cost are strong predictors [24]. By the simulation presented in Figure 4 and Figure 6, the medical healthcare cost increased with the increase of the previous year’s medical healthcare cost. As shown in Figure 3, healthcare costs were aggregated on the *x* or *y* axis. It indicates that healthcare costs were abruptly consumed. One reason may derive from the subscribers’ characteristics of the area-basis public health insurance. The proportion of low-income subscribers is higher than the other insurance [25]. In addition, the subscribers may not afford to spend the healthcare cost to maintain their health status.

As shown in Figure 2, about 1/3 of the subjects used the healthcare cost for musculoskeletal disorders. The number of subjects that used the healthcare cost for musculoskeletal disorders was higher than that of hypertension. In Japan, other than clinics of orthopedics, there are many treatment places managed by the bonesetter. These treatment places provide a massage, electric stimulation therapy, and hyperthermia treatment as rehabilitation. The national insurance system covers these treatments. It may be one of the reasons that many subjects used the healthcare cost for musculoskeletal disorders. The healthcare cost by kidney diseases was very high. More than JPY 10,000,000 per subject were used for the patients treated by hemodialysis. However, these patients were a tiny fraction. The prevention of kidney diseases by a high risk strategy may be a useful tool to reduce the healthcare cost.

There are many limitations to predict the healthcare cost by the conventional regression models. One of the solutions is to apply the zero-inflated model which consists of two components. However, this statistical model has a serious limitation. The variable that this model can deal with is a discrete variable. The neural network model can deal with both discrete and contentious variables. Our previous studies and the other study successfully predicted the medical healthcare cost by the neural network model [14,15,18]. Therefore, the neural network model as a nonlinear model may be an optimal statistical model to predict the healthcare cost.

When focusing on the axis of dental healthcare costs shown in Figure 6, spending the dental healthcare cost reduced the medical healthcare cost. However, its effect was a very tiny fraction. Periodontal disease was a risk for diabetes mellitus and diabetic complications: Diabetic retinopathy, neuropathy, nephropathy, cardiovascular complications, and mortality [26]. Oral health disorders were risks of hypertension [27]. The periodontal status affected hypertension [28]. Oral infections affected the prognosis of the patients with kidney disease [29,30]. The proportion of the medical healthcare cost of diabetes, hypertension, and kidney diseases was high (Figure 2A).

Poor oral hygiene has been suggested to be a risk for pneumonia, especially aspiration pneumonia [31]. Dental biofilm contains potential respiratory pathogens [32]. Oral hygiene behaviors including professional tooth cleaning by attending a dental clinic were associated with pneumonia [33]. Oral health intervention can reduce the incidence of pneumonia [34,35]. Therefore, using the dental healthcare cost is expected to reduce the healthcare cost for pneumonia. However, the study population of these studies were older adults. The subjects analyzed in this study were less than 75 years old. Insurance for the older adults over 75 years were different from the area-basis public health insurance. It is one of the limitations of this study. The prevalence of diseases and subsequent expenditure of healthcare cost may be different when limited to older adults. Older adults over the age of 65 spent four times the healthcare cost of the subjects less than 65 years old [1].

Improving the oral health status through dental treatment is expected to promote the health status and lead to the reduction of the medical healthcare cost. The Japanese insurance system covers not only the dental treatment, but also the supportive therapy by regular follow ups. However, when evaluating the health status by the medical healthcare cost, the dental treatment had an exiguous effect during a short period. The dental healthcare cost is low when compared to the medical healthcare costs. Oral health promotion affects the reducing prevalence of hypertension and type 2 diabetes, it will effectively act on reducing total healthcare costs. Therefore, a long term basis observational study is necessary to evaluate the effect of oral health on the medical healthcare cost. It is one of the limitations of this study. Electric data accumulation of the healthcare cost is just getting started in Japan.

## 5. Conclusions

The area-basis public health insurance database contains subjects with a wide range of age groups and their family members engaged in a variety of occupations. By analyzing this database, the robust statistical model for prediction can be obtained. Among the machine learning tools, the neural network model was the best method to predict the healthcare cost. The healthcare cost largely depended on the medical healthcare cost of the previous year. In addition, the dental treatment had an exiguous effect on the reduction of the medical healthcare cost.

## Figures and Tables

**Table 1:** Results of the regression model to estimate the medical healthcare cost.

**Independent Variable**	**Generalized Linear Model**		**Zero-Inflated Model**	
			**Zero-Inflation Model (Binomial, Link: Log-log)**	
			**Estimate**	***p*** **-Value**
(Intercept)			0.736	<0.001
Age			−0.0002	0.737
Sex (Women/Men)			−0.140	<0.001
Medical healthcare cost of the previous year			−0.259	<0.001
Dental healthcare cost of the previous year			−0.265	<0.001
			**Count model (Poisson, link: Log)**	
	**Estimate**	***p*** **-value**	**Estimate**	***p*** **-value**
(Intercept)	0.374	<0.001	1.390	<0.001
Age	0.004	<0.001	0.004	<0.001
Sex (Women/Men)	0.031	<0.001	−0.023	<0.001
Medical healthcare cost of the previous year	0.114	<0.001	0.058	<0.001
Dental healthcare cost of the previous year	0.077	<0.001	−0.003	0.462
AIC	178,481		130,725	

The zero-inflated model consisted of two components: For the hurdle model, zero hurdle model, and count model and for the zero-inflated model, zero-inflated model, and count model, respectively. The zero-hurdle model and zero-inflation model are models to estimate if the samples exceeded zero or not. The count model is a model to estimate if the optimal distribution of the samples exceeded zero. AIC: Akaike’s information criteria, the smaller AIC indicate the well model fit. The AICs of the models were very high.

## Data Availability

For the availability of data, approval of Ebina city council is necessary. If there is a reasonable reason, please ask the author Yoshimasa Ishii.

## Supplementary Materials

The following are available online at https://www.mdpi.com/1660-4601/18/2/565/s1. Figure S1: Relative influence of the factors for the medical healthcare cost by generalized boosted model; Table S1: Descriptive statistics of healthcare cost by age groups; Table S2: Information for the support vector machine regression model.
