# Supplementary material for: Does Last Year’s Cost Predict the Present Cost? An Application of Machine Leaning for the Japanese Area-Basis Public Health Insurance Database
Source: Int J Environ Res Public Health. 2021 Jan 12;18(2):565. doi: 10.3390/ijerph18020565 (PMC7827468; doi:10.3390/ijerph18020565)

International Journal of Environmental Research and Public Health

MDPI

Supplemental materials

Table 1. Descriptive statistics of health care cost by age groups.

	Age Group	n	Mean +/- SD (Japanese yen)	Median (25th-75th Percentile) (Japanese yen)
Medical health care cost	0-9	1104	168,764 +/- 668,363	55,215 (25,065 – 113,305)
	10-19	1467	86,823 +/- 475,628	26,080 (7,420 – 58,510)
	20-29	2059	77,938 +/- 409,758	12,080 (0 – 42,310)
	30-39	2594	157,601 +/- 2,098,765	15,040 (0 – 62,910)
	40-49	3773	241,190 +/- 12,886,38	20,200 (0 – 93,290)
	50-59	3242	330,496 +/- 1,201,088	38,560 (0 – 153,190)
	60-69	9268	424,605 +/- 1,250,412	113,985 (25,985 – 285,135)
	70-74	6833	545,069 +/- 1,467,711	187,700 (75,460 – 436,030)
Hospitalized patient care cost	0-9	1104	84,069 +/- 602,846	0 (0 - 0)
	10-19	1467	28,467 +/- 394,526	0 (0 - 0)
	20-29	2059	37,111 +/- 323,645	0 (0 - 0)
	30-39	2594	45,278 +/- 366,393	0 (0 - 0)
	40-49	3773	112,779 +/- 851,361	0 (0 - 0)
	50-59	3242	162,344 +/- 894,578	0 (0 - 0)
	60-69	9268	179,127 +/- 884,028	0 (0 - 0)
	70-74	6833	232,881 +/- 1,005,752	0 (0 - 0)
Outpatient care cost	0-9	1104	84,695 +/- 155,756	53,305 (24,330 – 106,140)
	10-19	1467	58,355 +/- 246,674	25,340 (7390 – 57,560)
	20-29	2059	40,828 +/- 177,899	11,460 (0 – 40,240)
	30-39	2594	112,323 +/- 2,061,664	14,605 (0 – 57,600)
	40-49	3773	128,411 +/- 919,462	19,420 (0 – 85,780)
	50-59	3242	168,152 +/- 668,661	35,495 (0 – 130,730)
	60-69	9268	245,478 +/- 745,560	107,270 (24,505 – 240,770)
	70-74	6833	312,188 +/- 926,886	172,210 (71,820 – 338,570)
Medication cost	0-9	1104	34,062 +/- 60,779	17,590 (5,775 – 41,130)
	10-19	1467	23,329 +/- 78,610	5,910 (0 – 20,350)
	20-29	2059	21,478 +/- 122,717	1970 (0 – 11,980)
	30-39	2594	37,791 +/- 117,176	2450 (0 – 17,230)
	40-49	3773	56,133 +/- 242,311	2240 (0 – 23,850)
	50-59	3242	66,410 +/- 238,501	3620 (0 – 44,360)
	60-69	9268	78,450 +/- 394,454	12015 (0 – 76,370)
	70-74	6833	99,374 +/- 303,106	25680 (0 – 115,330)
Dental health care cost	0-9	1104	20,010 +/- 30,286	6585 (0 – 31,680)
	10-19	1467	14,388 +/- 25,893	0 (0 – 21,160)
	20-29	2059	15,120 +/- 56,097	0 (0 – 13,970)
	30-39	2594	20,439 +/- 43,149	0 (0 – 22,530)
	40-49	3773	23,834 +/- 48,937	0 (0 – 29,520)
	50-59	3242	28,867 +/- 82,532	0 (0 – 37,260)
	60-69	9268	38,493 +/- 56,439	16,635 (0 – 57,940)
	70-74	6833	45,338 +/- 72,084	23,590 (0 – 68,880)

Medical health care cost is sum of hospitalized service cost and outpatient service cost. Data are sum of the cost from April 2017 to September 2018 (one year and half).

Table 2. Information for the support vector machine regression model.

**Number of Support Vectors**	**6245**
Objective Function Value	-273215.9
Training error	0.510993
Cross validation error	208078073376
Laplace distr. width	80939.37

Figure S1. Relative influence of the factors for the medical healthcare cost by model.

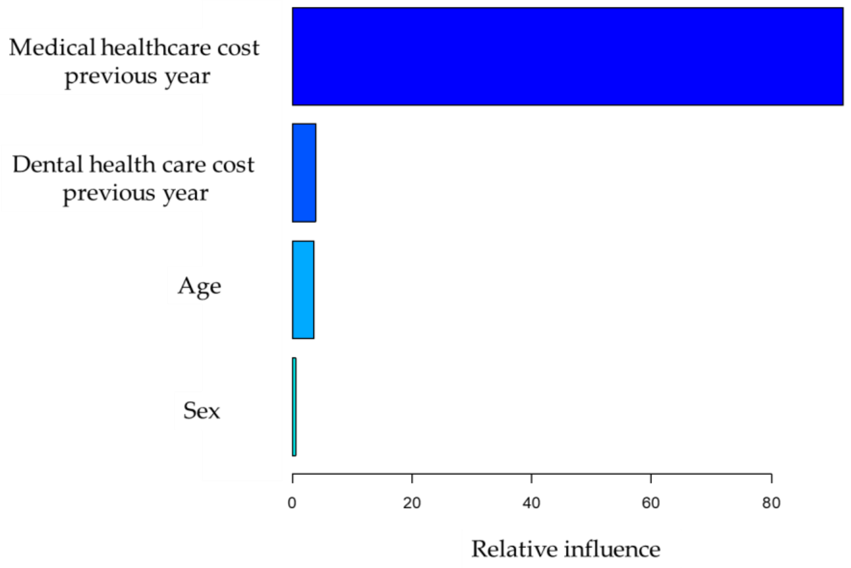

Supplement: Supplementary file 1 [file ijerph-18-00565-s001.pdf]
